# Confirmatory Factor Analysis Supports the Two-Factor Structure of the Arabic Fear-Avoidance Belief Questionnaire in Patients with Low Back Pain

**DOI:** 10.3390/healthcare13070800

**Published:** 2025-04-02

**Authors:** Ali H. Alnahdi, Mishal M. Aldaihan, Abdulrahman M. Alsubiheen

**Affiliations:** 1 Department of Rehabilitation Sciences, College of Applied Medical Sciences, King Saud University, P.O. Box 10219, Riyadh 11433, Saudi Arabia; mishaldaihan@ksu.edu.sa (M.M.A.); aalsubiheen@ksu.edu.sa (A.M.A.); 2 King Salman Center for Disability Research, Riyadh 11614, Saudi Arabia

**Keywords:** Kinesiophobia, fear of movement, psychometrics, patient-reported outcome measure, disability, fear-avoidance beliefs

## Abstract

**Background/Objective:** Inconsistencies exist regarding the exact multidimensional structure underlying the Fear-Avoidance Beliefs Questionnaire (FABQ), with no prior study examining the internal structure of the Arabic FABQ. This study aimed to examine validity evidence of the Arabic FABQ in patients with low back pain (LBP), based on two sources: validity evidence based on the internal structure (dimensionality and reliability) and validity evidence based on relations with other variables (i.e., pain intensity and disability). **Methods:** Participants (N = 112) with LBP were recruited from physical therapy clinics. Data were collected through the completion of FABQ and other measures of pain and disability. CFA was performed using a diagonally weighted least squares estimation. The fit of the two-factor model recommended by the original scale developer was assessed using multiple fit indices. Reliability of FABQ subscale scores was assessed using McDonald’s omega (ω) and Average Variance Extracted (AVE). **Results:** One hundred and twelve patients with LBP with mostly chronic complaints participated in the study. The CFA supported the two-factor model with modifications to account for residual correlations between items 4–5 and 6–7, yielding improved fit indices (χ^2^(41) = 77.82; *p* < 0.001; TLI = 0.98, CFI = 0.99, RMSEA = 0.09 (90% CI = 0.06–0.12), and SRMR = 0.08). All factor loadings were salient and significant with values ranging from 0.43 to 0.96. The two underlying factors reflecting physical activity-related and work-related fear avoidance beliefs showed a significant positive correlation of 0.58. These findings confirm the hypothesized dimensionality of the Arabic FABQ. The FABQ work subscale scores demonstrated higher reliability (ω = 0.86; AVE = 0.54) compared to the physical activity subscale scores (ω = 0.63; AVE = 0.44), with both factors measuring related but distinct constructs. The latent scores for the FABQ related to physical activity demonstrated stronger positive correlations with pain intensity (r = 0.37; *p* < 0.001) and disability (r = 0.43; *p* < 0.001), compared to the latent scores for work-related FABQ, which showed weaker correlations with pain intensity (r = 0.22; *p* < 0.001) and disability (r = 0.26; *p* < 0.001). **Conclusions:** This study provides evidence to support the two-factor structure of the Arabic FABQ and the common scoring method for the FABQ and facilitates the interpretation of the FABQ subscale scores as reflecting related but distinct domains of fear avoidance beliefs.

## 1. Introduction

Low back pain (LBP) is one of the most common musculoskeletal disorders and a leading cause of disability worldwide, affecting a substantial proportion of adults at some point in their lives [1,2]. While many individuals recover from acute episodes of LBP, a notable subgroup progresses to chronic LBP, experiencing persistent pain and disability that prove resistant to conventional therapeutic approaches [3]. The fear-avoidance model provides a conceptual framework for understanding why some patients transition to chronicity [4,5,6,7]. The fear-avoidance model has been widely applied to various chronic pain conditions beyond LBP, including osteoarthritis, fibromyalgia, chronic neck pain, and other chronic pain conditions where fear-avoidance beliefs contribute to disability and poorer outcomes [8]. According to this model, individuals who catastrophically interpret pain signals as threatening tend to develop maladaptive beliefs, leading to avoidance behaviors. This cycle of avoidance, deconditioning, increased pain sensitivity, and psychological distress perpetuates chronicity and hinders recovery.

The Fear-Avoidance Beliefs Questionnaire (FABQ), originally developed by Waddell and colleagues [9], is one of the most extensively used patient-reported outcome measures to quantify fear-avoidance beliefs in individuals with LBP. The FABQ consists of two subscales, one focusing on physical activity and the other on work-related fear-avoidance beliefs, allowing for a more comprehensive evaluation of how these beliefs impact daily functioning. Compared to other similar instruments, such as the Tampa Scale of Kinesiophobia (TSK) [10] and the Pain Catastrophizing Scale (PCS) [11], the FABQ is particularly relevant in clinical and occupational health settings due to its direct focus on work-related fear-avoidance behaviors [12]. While the TSK assesses general fear of movement and the PCS evaluates cognitive and emotional responses to pain, the FABQ provides a more targeted assessment of the behavioral consequences of these fears, making it especially valuable in return-to-work interventions and rehabilitation planning [13]. George et al. have demonstrated that FABQ scores significantly predict work disability and treatment outcomes, reinforcing its utility in clinical decision-making [14]. Thus, the FABQ remains a more relevant tool in assessing fear-avoidance beliefs, particularly in occupational and rehabilitation contexts. The psychometric properties of the FABQ have been documented across various cultures, demonstrating evidence of score reliability and validity evidence based on relations with related outcome measures [15,16,17,18,19,20,21,22,23,24,25,26]. Validity evidence based on internal structure (dimensionality) has also been investigated in different language versions of the FABQ, supporting a multidimensional construct reflected by the FABQ [15,16,17,18,19,20,21,22,23,24,25,27]. However, inconsistencies exist in the literature regarding the exact multidimensional structure underlying the FABQ, with some studies supporting a two-factor structure [15,16,17,18,19,20,27] while others suggest a different three-factor structure [21,22,23,24,25]. Most of these studies used exploratory factor analysis instead of the preferred hypothesis-driven method that is confirmatory factor analysis.

An Arabic version of the FABQ has been developed and tested, with studies suggesting acceptable evidence of scores, test-retest reliability, and validity evidence based on relations with related outcome measures in Arabic-speaking patients with LBP [28,29]. However, these investigations have not assessed validity evidence based on internal structure Arabic FABQ, including dimensionality and reliability. Without confirming its factor structure, the questionnaire’s utility may be limited, as the absence of established scale dimensionality could lead to misinterpretation of scores and suboptimal clinical decision-making. The lack of confirmed dimensionality of the Arabic FABQ questions the validity of the two-subscale scoring method for the Arabic FABQ and questions whether the subscale scores reflect the actual underlying structure of the Arabic FABQ [28,29].

The primary objective of this study is to examine validity evidence based on the internal structure (dimensionality and reliability) of the Arabic FABQ in patients with LBP using confirmatory factor analysis. We hypothesized that the Arabic FABQ will demonstrate a two-factor structure consistent with the structure reported in the original development of the scale. We also hypothesized that Arabic FABQ scores will demonstrate adequate reliability. The second goal is to assess the validity evidence based on relations with other variables, namely pain intensity and disability. Statistically significant correlations are expected between the latent scores of both FABQ dimensions and pain intensity and disability. By addressing this critical gap, the present study aims to strengthen the psychometric foundation of the Arabic FABQ, thereby improving clinical assessment and research endeavors in Arabic-speaking patients with LBP.

## 2. Materials and Methods

### 2.1. Study Design

This study used a cross-sectional design to examine validity evidence based on the internal structure (dimensionality and reliability) of the Arabic version of the FABQ in patients with LBP. Data were collected at a single point in time.

### 2.2. Setting and Participants

Participants were recruited from outpatient physical therapy clinics in the central region of Saudi Arabia within Alrass General Hospital and the Security Forces Hospital. A non-probabilistic convenience sampling approach was employed, inviting all patients who met the inclusion criteria and attended the participating clinics during the data collection period (June 2022 to June 2023) to enroll in the study. All participants were informed about the study’s purpose, procedures, and voluntary nature before providing written informed consent. Ethical approval for the current study was obtained from the ethics committee at security forces hospital (22-601-37) and King Saud university (E-20-5529).

Inclusion criteria for the current study were: (1) age ≥18 years; (2) referred to physical therapy clinics for LBP; and (3) sufficient reading comprehension in Arabic to complete the questionnaires independently. Participants were diagnosed by consultant physicians in orthopedic, spine, and primary health care clinics and subsequently referred to physical therapy. The criteria for exclusion in the current study encompassed: (1) systemic diseases (rheumatic conditions, metabolic disorders, autoimmune diseases, chronic infections, or malignancies affecting the spine); (2) cardiopulmonary or neurological conditions that functionally limit the participant; (3) musculoskeletal disorders distinct from LBP as identified by the patients to be a source of functional limitation; (4) spinal surgical interventions.

### 2.3. Procedure

At their initial visit to the physical therapy clinic and after verifying eligibility and obtaining written informed consent, participants completed the study questionnaires on-site, including the FABQ, Numeric Pain Rating Scale, and the Oswestry Disability Index. Demographic and clinical information related to the participants was also recorded.

### 2.4. Measures

The FABQ is a self-reported measure with 16 items that assess fear-avoidance beliefs in relation to physical activity (FABQ-PA) and work-related activities (FABQ-W) [9]. Each item is scored on a 7-point Likert scale ranging from 0 (“completely disagree”) to 6 (“completely agree”), with higher scores indicating stronger fear-avoidance beliefs. Scores for the FABQ-PA subscale (4 items: 2–5) range from 0 to 24, and those for the FABQ-W subscale (7 items: 6, 7, 9–12, 15) range from 0 to 42. Participants’ subscale scores were calculated by summing the relevant item scores. Items not included in subscale scoring were not used for the confirmatory factor analysis given that these items do not contribute to the FABQ subscales according to the original scale developer [9]. The item numbering followed the original publication; items 1, 8, 13, 14, and 16 were excluded, as recommended by the original authors of the instrument. The FABQ Arabic version scores have been reported to have sufficient test-retest reliability and validity evidence based on relations with related outcome measures but without prior validity evidence based on the internal structure (dimensionality) [28,29]. To provide a clear picture of the clinical presentation of the participants, pain intensity was measured using the Numeric Pain Rating Scale (NPRS) (score: 0–10), and low back-related disability was measured using the Oswestry Disability Index (ODI) (score: 0–100), with higher scores indicating greater pain intensity and greater disability. Prior studies supported the measurement properties of the Arabic versions of these measures [30,31,32].

### 2.5. Statistical Analysis

#### Confirmatory Factor Analysis (CFA)

To evaluate the internal structure (dimensionality and reliability) of the Arabic FABQ, a CFA was conducted. The CFA model tested included a two-factor model that was recommended by the original scale developer [9]. The first latent factor represented fear avoidance beliefs related to physical activity with four indicators (items 2, 3, 4, 5), and the second latent factor represented fear avoidance beliefs related to work with seven indicators (items 6, 7, 9, 10, 11, 12, 15). Given that the FABQ items use a Likert-type scale with ordinal data and that multivariate normality needed for maximum likelihood estimation was violated as indicated by mardia’s test, a diagonally weighted least squares (DWLSs) estimation method with robust standard errors was used [33,34]. DWLS is recommended for ordinal data because it does not assume continuous or normally distributed variables and is more robust to violations of normality [33,34,35]. Fit indices for evaluating model fit, such as the chi-square statistic (χ^2^), the ratio of χ^2^ to degrees of freedom, the Tucker-Lewis index (TLI), the comparative fit index (CFI), the standardized root mean square residual (SRMR), and the root mean square error of approximation (RMSEA), were used to gauge the fit of the specified model. Regular versions of the fit indices were used. TLI and CFI values of no less than 0.95, an RMSEA of 0.06 or lower, and an SRMR of 0.08 or lower were indicative of an adequately fitting model [36,37,38]. Instances of model misspecification were detected through the analysis of standardized residuals and modification indices, which highlight areas of potential enhancements for the model [35,39]. The reliability of the FABQ scores was assessed using McDonald’s ω and the Average Variance Extracted (AVE) for the physical activity and work subscales (FABQ-PA; FABQ-W). McDonald’s ω was chosen as it provides a more robust estimate of score reliability (internal consistency) compared to Cronbach’s α, particularly when factor loadings are not tau-equivalent [40,41].

After achieving a satisfactory fit for the FABQ measurement model, NPRS and ODI were added as observed variables in the structural equation model (SEM) to investigate the relationships between the latent scores of the FABQ-PA and FABQ-W with pain intensity measured by NPRS and disability measured by ODI. The estimation methods and model fit assessment employed for the measurement model were also applied for the full SEM model. All statistical analyses were conducted using JASP (Version 0.19.1) [42] and Jamovi (Version 2.6.26) [43]. The CFA analysis in JASP and Jamovi is built on the Lavaan R Package for structural equation modeling [44].

### 2.6. Sample Size Estimation

The sample size needed for the CFA analysis was estimated using the “semPower.aPriori” function within the semPower R package [45]. The necessary sample size for identifying misspecifications of a model that includes 41 degrees of freedom, an RMSEA effect size of 0.08, with α set at 0.05 and a statistical power (1 − β) of 0.80. The estimated sample size was 108 participants.

## 3. Results

### 3.1. Sample Characterization

Participants in the current study were 112 patients with LBP (Table 1). Participants had, on average, moderate pain intensity and moderate perceived disability as indicated by their average score in the Numeric Pain Rating Scale and the Oswestry Disability Index. None of the participants had any missing data in the FABQ items thus, no imputation was performed.

### 3.2. Validity Evidence Based on Internal Structure: Dimensionality

**Figure 1:**
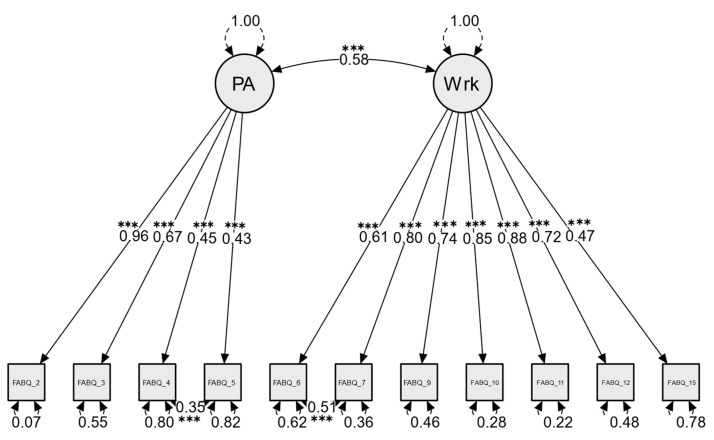
FABQ CFA measurement model with two correlated latent factors representing fear avoidance beliefs about physical activity (PA) and fear avoidance beliefs about work (Wrk). The model accounted for correlated measurement error between items (4, 5) and (7, 8). Curved, double-headed arrows drawn at each item represent the residual variance, indicating the percentage of each item’s variance that remains unexplained by the latent factor. *** *p* < 0.001.

The first CFA model tested included a two-factor model that was recommended by the original scale developer [9]. The first latent factor represented fear avoidance beliefs related to physical activity with four indicators (items 2, 3, 4, 5) and the second latent factor represented fear avoidance beliefs related to work with seven indicators (items 6, 7, 9, 10, 11, 12, 15). This CFA model yielded the following fit indices: χ^2^(43) = 104.37, *p* < 0.001, TLI = 0.97, CFI = 0.98, RMSEA = 0.11 (90% CI = 0.09–0.14, *p*[RMSEA ≤ 0.05] < 0.001), and SRMR = 0.10. These results suggest that the model was close to an acceptable fit to the two-factor structure. Examination of modification indices highlighted specific points of misfit, particularly elevated error covariance between the following item pairs (items 6, 7) and (items 4, 5). Allowing residual covariance among these pairs of items, as illustrated in Figure 1, significantly improved the fit of the model (Δχ^2^ = 26.5; Δdf = 2; *p* < 0.001) and yielded improved fit indices: χ^2^(41) = 77.82, *p* < 0.001, TLI = 0.98, CFI = 0.99, RMSEA = 0.09 (90% CI = 0.06–0.12; *p*[RMSEA ≤ 0.05] = 0.021), and SRMR = 0.08. These fit indices suggested a good fitting model after the modifications were implemented (Figure 1). The FABQ-PA items showed significant positive correlation with their respective latent factor, ranging from 0.43 for item 5 to 0.96 for item 2, while the FABQ-work items showed significant positive correlation with their respective latent factor, ranging from 0.47 for item 15 to 0.88 for item 11 (Figure 1). The CFA employed also estimated the latent correlations between factors and residuals. The two underlying factors representing fear avoidance beliefs about physical activity and work showed a significant positive correlation (r = 0.58, *p* < 0.001) (Figure 1). Similarly, significant positive correlation was determined between the residuals of item pairs (items 4, 5; r = 0.35, *p* < 0.001) and (items 6, 7; r = 0.51, *p* < 0.001) (Figure 1).

### 3.3. Validity Evidence Based on Internal Structure: Reliability

The reliability assessment of the FABQ subscale scores revealed that McDonald’s ω for the FABQ-PA scores was 0.63, while the FABQ-W subscale scores demonstrated higher internal consistency of 0.86. The AVE for the FABQ-PA was 0.441, indicating that less than half of the variance in the items was explained by the latent construct. In contrast, the AVE for the FABQ-W subscale was 0.544, suggesting relatively better score reliability. Both the FABQ-PA (0.441) and FABQ-W (0.544) factors have AVE values greater than the squared correlation between PA and Work (0.577^2^ = 0.333), this indicates that both FABQ factors (subscales) demonstrated evidence of measuring related but distinct constructs [46].

### 3.4. Validity Evidence Based on Relations with Other Variables

The full SEM model that contains the FABQ measurement model and the correlation between the latent scores of the FABQ-PA and FABQ-W and NPRS and ODI reached an acceptable fit χ^2^(59) = 114, *p* < 0.001, TLI = 0.97, CFI = 0.98, RMSEA = 0.09 (90% CI = 0.06–0.12; *p*[RMSEA ≤ 0.05] = 0.005), and SRMR = 0.09. The latent scores of the FABQ-PA showed significant positive correlation with NPRS (r = 0.37, *p* < 0.001) and ODI (r = 0.43, *p* < 0.001). The latent scores of the FABQ-W showed significant positive correlation with NPRS (r = 0.22, *p* < 0.001) and ODI (r = 0.26, *p* < 0.001). Additionally, the latent scores of the FABQ-PA and FABQ-W demonstrated significant positive correlation (r = 0.48, *p* < 0.001).

## 4. Discussion

The present study investigated the factor structure of the Arabic version of the FABQ using Confirmatory Factor Analysis. Our main finding was that a two-factor model corresponding to fear-avoidance beliefs in relation to physical activity (FABQ-PA) and fear-avoidance beliefs in relation to work-related activities (FABQ-W) provided a good fit to the data. This result supports our primary hypothesis, which posited that the Arabic FABQ would corroborate the original two-factor structure. The outcome of the current study reinforces the conceptualization of fear-avoidance beliefs as comprising two distinct but related domains. Therefore, the current analysis provides validity evidence based on the internal structure (dimensionality and reliability) of the Arabic FABQ in a sample of patients with LBP.

The CFA results indicated that the pre-specified two-factor solution adequately accounted for covariation among the Arabic FABQ items, with robust factor loadings and acceptable model fit statistics (CFI, TLI, RMSEA, SRMR). These results imply that the Arabic FABQ items load distinctly onto two latent constructs—fear-avoidance beliefs related to physical activity and those related to work—consistent with the underlying theoretical framework of the instrument [9]. Similar to the analysis of the current study, a number of previous studies examined the validity of the correlated two-factor structure proposed by the original developer of the scale using confirmatory factor analysis [17,19,27,47]. The authors of these FABQ versions concluded an acceptable fit of the original two-factor model. Examining the results of these studies suggests that our study demonstrated better fit as indicated by the multiple fit indices compared to the Japanese FABQ [27] (Goodness of Fit Index (GFI) = 0.84, Adjusted GFI (AGFI) = 0.76), Chinese FABQ [19] (TLI = 0.81, CFI = 0.85, SRMR = 0.08, GFI = 0.86, AGFI = 0.79), German FABQ [17] (RMSEA = 0.09), and the Dutch FABQ [47] (CFI = 0.93, RMSEA = 0.07, GFI = 0.98). The authors of these versions did not report the specific estimation method used except for the Dutch version [47], where the authors employed weighted least squares estimation.

Several previous FABQ investigations examined the scale factor structure using Exploratory Factor Analysis (EFA), producing conflicting or inconclusive factor solutions [15,16,18,20,21,22,23,24,25]. The variability in these findings may stem from differences in sample characteristics (e.g., acute vs. chronic pain populations, sample size, and clinical settings) or methodological limitations. Moreover, EFA methods can be more susceptible to over-extraction or under-extraction of factors based on rotation techniques, eigenvalue cutoffs, and the subjectivity in interpreting scree plots [48]. The limitations of EFA are documented, particularly its inability to confirm theoretical models [34,35]. Consequently, the lack of a consistent factor solution in some prior FABQ studies has hindered confidence in the questionnaire’s structural validity. By applying CFA, we imposed a theoretically driven model (i.e., the established two-factor FABQ structure) on the data to verify its suitability. This approach, supported by strong empirical evidence for the FABQ in multiple cultural contexts, provides a more rigorous test of the instrument’s dimensionality and reduces some of the ambiguity seen in earlier EFA-based research. This approach aligns with the recommendation of COSMIN guidelines [48], ensuring robust and reproducible findings.

In our model, two pairs of items (4, 5) and (6, 7) exhibited notable residual covariances, meaning that after accounting for the common latent factor, these item pairs shared variance above and beyond what the model predicted. One potential explanation is semantic overlap or redundancy in the item content. This overlap can occur when two items address highly similar aspects of fear-avoidance, prompting respondents to score them in a similar manner. The observed residual correlations between items 4–5 and 6–7 are likely due to semantic similarity in their phrasing. Items 4 and 5 both assess physical activity-related fear-avoidance beliefs, with item 4 stating, “I should not do physical activities that (might) make my pain worse”, and item 5 stating, “I cannot do physical activities that (might) make my pain worse”. The minor difference may not be strongly differentiated by respondents, leading to similar response patterns and residual correlation. Similarly, items 6 and 7, which assess work-related fear-avoidance beliefs, exhibit conceptual overlap. Item 6 states, “My pain was caused by my work or by an accident at work”, while item 7 states, “My work aggravated my pain”. While item 6 attributes the initial cause of pain to work, item 7 suggests work as a factor that worsens existing pain. This distinction may not be sufficiently clear to respondents, contributing to their correlated responses. While such residual correlations do not necessarily compromise the integrity of the two-factor model, they highlight the need to ensure that questionnaire items remain clinically distinct and non-repetitive. Swinkels-Meewisse et al. reported high residual correlation between items 6 and 7 in their correlated two-factor FABQ model. This residual correlation was added and maintained in their final two-factor model [47]. The findings of this study are consistent with the residual correlation pattern reported in our study. The authors of several studies that used CFA to examine the underlying structure of the FABQ did not report any assessments related to model misspecification, deviations, or any potential modifications to the FABQ [17,19,27]. Thus, we have no information related to whether high residual correlations existed among FABQ items in these studies.

The moderate correlation (0.58) between FABQ-PA and FABQ-W subscales supports the conceptual distinction between fear-avoidance beliefs related to physical activity and work, while also highlighting their interrelated nature. This is consistent with findings from Pei et al. in the Chinese FABQ [19] and Swinkels-Meewisse et al. in the Dutch FABQ [47], which reported correlations ranging from 0.30 to 0.37. The observed correlation reflects potentially shared underlying cognitive mechanisms, such as pain catastrophizing, that influence both domains [4]. Clinically, the distinct yet correlated nature of these factors underscores the importance of addressing both domains in comprehensive treatment plans. For instance, interventions targeting physical activity beliefs should focus on reducing fear and promoting movement, while those targeting work-related beliefs may require collaboration with employers to implement supportive measures.

The scores reliability results indicate that the FABQ-W subscale scores exhibit strong internal consistency and satisfactory convergent validity, as evidenced by a high ω value and an AVE above the recommended threshold [46]. These findings align with previous research that has reported similar reliability coefficients for the work subscale [9,18]. In contrast, the FABQ-PA subscale scores demonstrated lower reliability, suggesting moderate internal consistency, and an AVE below 0.50, which indicates that the construct may not be well captured by its items. This finding is consistent with previous studies reporting lower reliability for the FABQ-PA subscale scores compared to the FABQ-W) [17,19,27]. The reliability (internal consistency) estimates reported in the literature were based on Cronbach’s α rather than the more appropriate statistics ω reported in the current study. The lower AVE for the FABQ-PA subscale suggests that additional refinement may be needed. It is important to note that none of the previous studies that examined the FABQ using CFA reported the AVE for the FABQ subscales [17,19,27,47], thus no comparisons can be made. These results highlight the need for further psychometric evaluation of the physical activity subscale to enhance its measurement properties and ensure its applicability in clinical and research settings. The findings confirm that both FABQ factors (subscales) demonstrated evidence of measuring related but distinct constructs [46], as the AVE values for PA and work factors are greater than the squared latent correlation. This suggests that fear avoidance beliefs about physical activity and fear avoidance beliefs about work are conceptually distinct constructs, supporting the adequacy of the measurement model.

The full SEM model demonstrated an acceptable overall fit to the data, supporting the proposed measurement and structural paths between the FABQ domains and the pain and disability measures. The FABQ-PA and FABQ-W domains of the FABQ showed significant positive correlations with both pain intensity (NPRS) and disability (ODI), consistent with the theoretical framework of the fear-avoidance model of chronic pain [4]. The FABQ-PA latent scores were more strongly correlated with both NPRS and ODI than the FABQ-W latent scores. This finding suggests that fear-avoidance beliefs related to general physical activity have a stronger connection with both pain intensity and disability than work-specific fears in this population. Using the Arabic FABQ, Laufer et al. reported that in patients with chronic LBP, measures of pain intensity correlate more strongly with FABQ-PA compared to the FABQ-W [29]. This pattern is consistent with the findings of our study, given that the majority of the participants in our study had chronic complaints. In line with our findings, low back-related disability was reported to have a higher correlation with FABQ-PA compared to FABQ-W in a Dutch sample with LBP [47]. Alanazi et al. reported low correlation between the scores of the Arabic FABQ and measures of pain intensity and disability, but these relations were not examined separately for each FABQ domain and thus cannot be directly compared to our findings [28]. To the best of our knowledge, the relations between the two latent factors representing the subscales of the FABQ with pain intensity and disability measures reported in the current study in patients with LBP have not been reported previously within a SEM framework.

Providing validity evidence for the Arabic FABQ has important clinical implications for treatment strategies in the Arab-speaking world, where culturally specific beliefs and attitudes toward pain and activity may influence recovery. With a psychometrically sound tool, clinicians can reliably assess fear-avoidance beliefs and use this information to tailor interventions that address both physical and psychological barriers to recovery. For example, patients with high FABQ scores may benefit from cognitive-behavioral strategies, pain education, and graded activity programs aimed at reducing fear and improving function. In contrast, lower scores may suggest that standard physical rehabilitation approaches are sufficient. By integrating the FABQ into routine assessment, clinicians can identify individuals at risk of prolonged disability and apply a more targeted, biopsychosocial treatment approach, ultimately improving outcomes in Arabic-speaking patients with low back pain.

A number of limitations in the current study need to be acknowledged. First, the sample was limited to patients from physical therapy clinics in Saudi Arabia, which may restrict generalizability to other Arabic-speaking populations. Future research should examine the FABQ in broader clinical and occupational settings. Second, although CFA confirmed the two-factor structure, Item Response Theory (IRT) analysis could provide deeper insights into item functioning, including response options behavior and differential item functioning (DIF) across subgroups. Future studies should explore IRT methods to refine the scale further. Third, measurement invariance was not tested across key subgroups (e.g., acute vs. chronic LBP, employed vs. unemployed). Multi-group CFA in future research could determine whether the FABQ is interpreted consistently across different populations. Finally, as this study was cross-sectional, it could not assess FABQ stability over time. Longitudinal studies are needed to examine predictive validity and factor structure stability, particularly in relation to clinical outcomes and treatment response.

## 5. Conclusions

This study provides evidence to support the two-factor structure of the Arabic FABQ and the common two subscale scoring method for the FABQ and facilitates the interpretation of the FABQ subscale scores as reflecting related but distinct domains of fear avoidance beliefs (physical activity-related and work-related) with fear-avoidance beliefs related to physical activity more strongly associated with both pain intensity and disability than work-related fears. The reliability of the physical activity subscale needs further assessment with considerations of possible refinement to enhance reliability. The validity evidence based internal structure presented in the current study enhances the Arabic FABQ’s utility in clinical and research settings. This study provided validity evidence-based on relations with other variables (pain intensity and disability), further supporting the utility of the Arabic FABQ in assessing fear-avoidance beliefs in patients with LBP. These findings contribute to the psychometric foundation of the FABQ, ensuring its robustness in assessing fear-avoidance beliefs in the Arabic-speaking population with LBP.

## Figures and Tables

**Table 1:** Characteristics of participants (N = 112).

Variable	Mean ± SD or N (%)
Age (year)	31.13 ± 12.49
Sex	
Male	53 (47.3)
Female	59 (52.7)
Height (m)	1.67 ± 0.10
Mass (Kg)	71.48 ± 12.18
Body mass index (Kg/m^2^)	25.98 ± 5.63
Work Status	
Employed	72 (64.29)
Housework	20 (17.86)
Unemployed	18 (16.07)
Retired	2 (1.79)
LBP duration	
Acute (<1 month)	16 (14.29)
Subacute (1–3 months)	19 (16.96)
Chronic (>3 months)	77 (68.75)
FABQ-PA (0–24)	15.27 ± 5.53
FABQ-W (0–42)	17.38 ± 10.55
ODI (0–100)	25.96 ± 15.55
NPRS (0–10)	5.54 ± 2.04

LBP = Low back pain; FABQ-PA = Fear-avoidance beliefs questionnaire-physical activity subscale; FABQ-W = Fear-avoidance beliefs questionnaire-work subscale; ODI = Oswestry disability index; NPRS = Numeric pain rating scale.

## Data Availability

The data that support the findings of this study are available from the corresponding author, upon reasonable request.
